# Persistence of Internal Representations of Alternative Voluntary Actions

**DOI:** 10.3389/fpsyg.2013.00202

**Published:** 2013-05-06

**Authors:** Elisa Filevich, Patrick Haggard

**Affiliations:** ^1^Institute of Cognitive Neuroscience, University College LondonLondon, UK; ^2^Max Planck Institute for Human Development, Max Planck Institut für BildungsforschungBerlin, Germany

**Keywords:** free action, response selection, Hick’s law, volition, reselection

## Abstract

We have investigated a situation in which externally available response alternatives and their internal representations could be dissociated, by suddenly removing some action alternatives from the response space during the interval between the free selection and the execution of a voluntary action. Choice reaction times in this situation were related to the number of initially available response alternatives, rather than to the number of alternatives available effectively available after the change in the external environment. The internal representations of response alternatives appeared to persist after external changes actually made the corresponding action unavailable. This suggests a surprising dynamics of voluntary action representations: counterfactual response alternatives persist, and may even be actively maintained, even when they are not available in reality. Our results highlight a representational basis for the counterfactual course of action. Such representations may play a key role in feelings of regret, disappointment, or frustration. These feelings all involve persistent representation of counterfactual response alternatives that may not actually be available in the environment.

## Introduction

Voluntary action involves selection of one action alternative amongst a series of equally available ones. Rapidly changing environments may impose sudden changes to the set of effectively available alternatives. Imagine a field hockey player running toward the goal with a ball, and coming to face the goalkeeper. Whilst she is deciding whether to push the ball right or left to the goalkeeper, a defense player suddenly comes to block the left side of the goal, leaving the attacker with only one effective alternative (pushing the ball to the right) if she wants to get the goal.

Several components can be identified in these situations of action selection. First, an internal response space must be constructed, containing representations of possible alternative responses (Fletcher et al., 2000). Next, one response (Gold and Shadlen, 2007) must be selected from the response space. Finally, the corresponding action must be prepared and executed (Deecke et al., 1969). Clearly, these processes may be dynamically updated with changes in the environment. For example, the motor plan may need to be adjusted, or completely switched after it has been selected (Wise and Mauritz, 1985; Snyder et al., 2000; Resulaj et al., 2009). A final step in the process is often neglected: the representations of the non-selected (or counterfactual) response alternatives must be dismantled (Logan et al., 1984).

Early views of action selection considered these processes to occur serially (e.g., Keele, 1968). However it is now widely recognized that these are not independent processes, and that they instead occur in parallel, and influence each other by competitive inhibition (Cisek, 2007; Cisek and Kalaska, 2010). For example, there is converging evidence suggesting that action selection is in play until relatively late in the chain of events, and that it co-occurs with action preparation (Sakai et al., 2000; Cisek and Kalaska, 2005; Klein-Flügge and Bestmann, 2012). Importantly, parallel processing models are not consistent with the notion of a single, definitive process of action selection. Rather, multiple action representations may persist with different levels of activation, through an extended period of preparation, until one dominant action emerges from the competition. On this view, an action may be actively entertained even if it is not the “front-runner” in the response selection process, and ends up being counterfactual. This parallelism of voluntary action representations could allow for adequate flexible behavior in a dynamic environment. Suppressing a response alternative too early may make it harder to reactivate it if circumstances require. A concomitant disadvantage of parallelism is that non-selected alternatives may remain needlessly activated.

Unselected, counterfactual action representations have proved difficult to study for the simple methodological reason that they have no behavioral output. Therefore, most current knowledge comes from animal studies where it is sometimes possible to record directly the neural signals involved in decision processes (Cisek and Kalaska, 2005), or from human imaging studies where the relative reward value of the non-chosen alternative should be tracked (Boorman et al., 2011; Rushworth et al., 2011).

We have developed an indirect, behavioral measure of counterfactual action based on the number of alternatives in the response space. In a choice reaction task, the reaction time depends strongly on the number of potential response alternatives (response set size). Hick (1952) found monotonic, increasing relations between reaction times (RTs) and set size, now widely known as “Hick’s law.” Hick’s Law is often explained by the additional time required to compare a stimulus repeatedly with each entry in a stimulus-response look-up table, and thus retrieve the correct action from the response space. Importantly, a crucial distinction must be made, between *external* and *internal* response sets – or “in the world” and “in the brain” c.f. (Gold and Shadlen, 2007). The former refers to the response alternatives that are effectively available in the external environment. The latter refers to the internal representations of the alternatives within the response space. While the external and internal sets should normally match, they need not do so, and only internal set sizes can influence RTs. This possibility allows us to test whether an unselected and unexecuted action is nevertheless represented within the internal response set. In particular, a higher RT than the external set size would predict could potentially be explained by the presence of an additional, counterfactual action representation within the response space.

Hick’s law has classically been applied to instructed actions, where a stimulus explicitly tells participants which action to make in every trial. In voluntary actions, by contrast, there are no explicit instructions about which action to make, and the participant instead freely selects one action from the response space. The underlying neural structures of voluntary actions differ from those for instructed actions (Krieghoff et al., 2011). In particular, voluntary action, but not instructed action, in parallel models of action selection may lead to feelings of regret (Boorman et al., 2009). Feelings of regret can be defined as a negative value in the comparison between the outcomes of the chosen alternative with the counterfactual alternative (Coricelli et al., 2005). To make this comparison, the relative value of the chosen and un-chosen alternatives should be computed. Therefore, some representation of the counterfactual alternatives must remain until after the choice was made. Thus, regret implies the persistent representation of actions that were included in the response space, but were not in fact selected.

Here, we asked whether internal response sets retain traces of counterfactual action when the external response set changes, by using an experimental analog of the hockey goal shooting example mentioned at the start of this article. The task necessitated three main features. First, it should present participants with a dynamic response space, in which some freely selected alternatives might suddenly become unavailable. Second, the task should allow the initially selected (but subsequently inhibited) response to be identified. For this aspect, we relied on participant’s subjective reports made after the trial. Third, and crucially, the task had to provide an implicit behavioral measure demonstrating the covert representation of unexecuted alternative actions, without explicitly reactivating them.

## Materials and Methods

### Participants

Eighteen naïve participants (11 female, mean age ± SD; 24 ± 5 years) took part in the study. One participant did not update their choice following changes in the number of available alternatives, and in fact selected disappeared locations. Their data was therefore excluded from the analysis. This yielded a total of 17 participants. All participants had normal or corrected to normal vision. Procedures were approved by the University College London research ethics committee and were in accordance with the principles of the Declaration of Helsinki.

### Task

**Figure 1:**
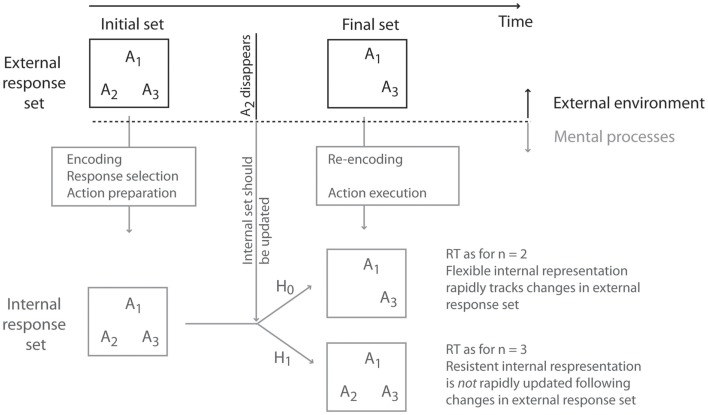
**Rationale and hypothesis of the study**. An initial external response set suddenly changes (in the example of the figure, response alternative A2 suddenly becomes unavailable). In these cases, the internal representation of the response alternatives must be updated to reflect the changes in the external environment. Two scenarios are thus possible. The internal response set may be flexible (Null hypothesis: H_0_), and can update its contents to respond rapidly to external changes. Alternatively (Experimental hypothesis: H_1_), the internal response set may be resilient to change, and the internal response set may lag behind changes in the external environment.

We asked participants to voluntarily select a response from an initially available set of responses. Once an intentional decision had been made, but before the response was executed, the external response set was suddenly reduced. On some trials, therefore, the response that the participant had already selected would become unavailable, and they would need to reselect another, alternative response from the updated external response set. Using RTs as a proxy for the internal response set size, we addressed whether the internal response set had been rapidly updated to match the new external response set, or whether the internal response set lagged behind the external changes (see Figure 1). Two scenarios were possible. In the first place, the internal representation of the response alternatives could perfectly track the external response set. Alternatively, the internal representation of the response alternatives could contain a persistent representation of the initially selected and now unavailable response.

Because the size of the response set is different, the two cases can be distinguished using Hick’s Law, even if they are behaviorally identical. If initially selected response alternatives were effectively removed from the internal response set once they become unavailable, RTs would increase as a function of the final response set size (and not the initial response set size). Conversely, if the neural representation of the initially selected but now unavailable response is maintained in the internal response set, then RTs would increase as a function of the initial response set size.

Stimuli were displayed on a CRT monitor with a refresh rate of 60 Hz. Participants sat 60 cm away from the screen. The experiment consisted of six blocks of 100 trials and lasted for approximately 50 min. Each trial belonged to one of four experimental conditions that will be described thoroughly below. These were *no change* (34% of the total number of trials), *instructed selection* (20%), and *original selection* and *reselection* (together, 46%). The exact proportion was partly determined by the participants’ behavior, see below.

**Figure 2:**
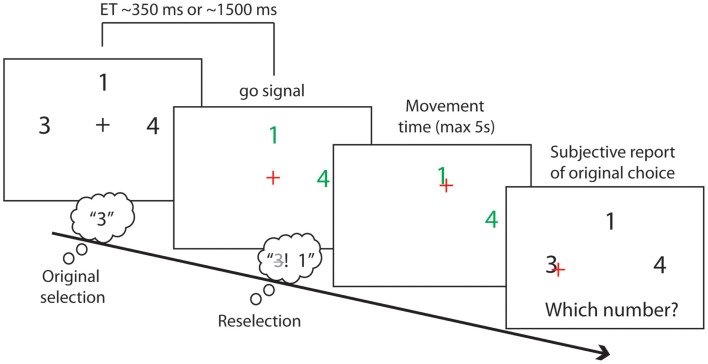
**Experimental task**. The initial response set of numbers was presented for either a short (350 ± 200 ms) or a long (1500 ± 200 ms) exposure time (ET). Participants covertly selected a number during the exposure time. A change of fixation color indicated the go signal. Participants could then move a cursor to click on a number of their choice. At the same time as the go signal onset a subset of the presented numbers could disappear, leaving a final response set with a size between 1 and the original response set size. Participants could click on their originally chosen number if it remained in the final response set. Instead, they would have to reselect a number other than their first choice if it had disappeared from the final response set. Participants then reported their original number choice.

At the start of each trial, one to four different numbers were displayed on the screen, arranged around a central fixation cross with 2° eccentricity (see Figure 2). Number location and identity were randomized. All stimuli were displayed over a black background. We used numbers as targets because we sought to minimize the working memory load on both target selection and recall, minimizing in turn the problems and potential biases associated with subjective report.

The set of numbers first presented in each trial was the initial response set. Numbers in the initial response set were randomly sampled without repetition from the numbers 1–9 excluding the number 5 (see *instructed* condition below). The numbers in the initial response set were displayed in white for either a short or a long exposure times (ETs). Short ETs were periods of 550 ms with a random jitter of a maximum of ±200 ms. Long ETs were periods of 1500 ms with random jitters of a maximum of ±200 ms. Participants were asked to covertly select one of the numbers in the initial response set during the ETs, and to prepare to move a cursor and click on the number using a large trackball mouse (Keytools Ltd., Southampton, UK). They were instructed to make a new free selection on each trial, avoiding stereotyped responses or sequential patterns. The ETs was varied to allow more or less time for this initial selection process. Short and long ETs were randomly assigned to experimental trials. We assumed that longer ETs would allow for stronger action preparation and a stronger, and therefore more persistent, encoding of the initial response set.

After the ETs, the fixation cross changed color, from white to red. This was the “Go” signal, instructing participants to move to the selected target number. Crucially in the *original selection* and *reselection* conditions, a subset of the numbers in the initial response set disappeared at the time of the Go signal. The remaining numbers changed color and turned to green. The number of disappearing targets varied from zero to *n* − 1, where *n* is the initial response set size. Consequently, the *final* response set varied from one item to the full initial response set. The positions and identity of the disappearing numbers were fully randomized.

After the go signal, participants moved the trackball to bring the cursor to the selected number, and clicked the mouse button. They were instructed to make this action as quickly and as rapidly as possible. If the originally chosen number had disappeared from the final response set, participants were asked to reselect a different number, from the smaller final response set of available alternatives. Otherwise, they were to execute the originally selected response.

**Figure 3:**
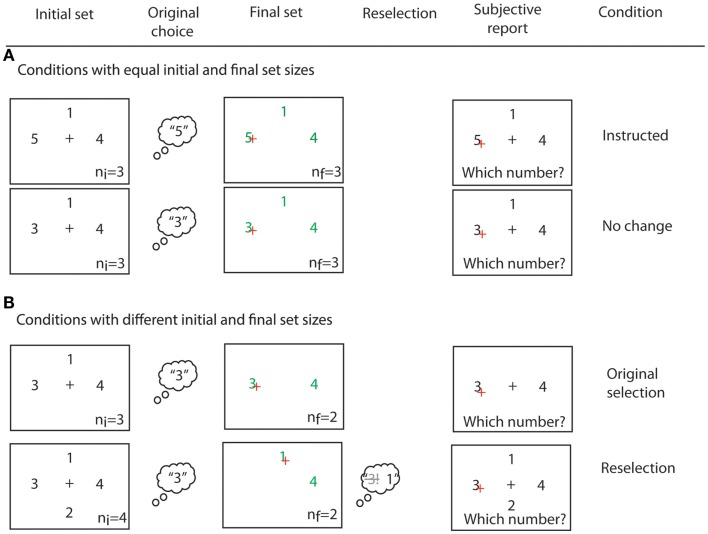
**Examples of all four experimental conditions**. **(A)** Conditions with equal initial and final response set sizes. If a number “5” was present in the initial response set, participants were instructed to click on it (*instructed* condition). In the *no change* condition, the number choice was intentional. **(B)** Conditions with non-matching initial and final response set sizes. In the original selection and reselection conditions, some numbers disappeared from the initial response set. A trial was sorted as *original selection* if participants reported that their original choice matched their final choice. Instead, a trial was sorted as *reselection* if participants reported having chosen a number that had become unavailable. *n*_i_ and *n*_f_ indicate the initial and final response set sizes, respectively. They were not displayed in the experiment.

After clicking on the target number, participants reported which number they had *originally* chosen in all conditions, and regardless of which number they had clicked on. In this way, trials in which reselection had occurred could be identified on the basis of subjective report. We assumed that reselection had occurred if the reported original choice did not match the clicked number, and if the original choice had disappeared. Otherwise, trials were classified as “simple selection” (see Figure 3B). At debriefing, no participant reported difficulties in the report of their original choice.

Our task crucially required that participants did indeed select from the initial response set, rather than simply wait for the appearance of the final response set, as only then could we evaluate the persistence of suddenly unavailable response alternatives. We used two strategies to ensure that participants attended to the initial response set, and selected an action from it. First, we included *instructed* trials. If the number “5” was found in the initial response set, participants were instructed to always select this number, and execute the corresponding response on seeing the Go signal. No numbers were removed from the initial response set in *instructed* trials. Second, to prevent participants from simply waiting for the final response set, we included a *no change* condition, in which the Go signal appeared but no numbers were removed from the initial response set (see Figure 3A).

To encourage action preparation following the initial response set, and therefore inhibition of the prepared response, we rewarded participants for quick responses (measured as time to click on the target relative to the onset of the go signal, i.e., the sum of reaction time and movement time). We informed participants that they would get 0.5 p extra for every trial that was quicker than their own average in the preceding block. Therefore, the experimental design discouraged the potential strategy of ignoring the initial response set completely and waiting for the final response set instead. Participants earned on average £ 2.23 (±SD £0.03).

To discourage participants from adopting a predetermined choice strategy, both the identity and the spatial location of the targets varied randomly from trial to trial. Randomly sampled numbers were displayed on the vertices of a square with an angular tilt of either 0° or 90°. The position of the targets was fully randomized.

Importantly, the initial and final response set sizes were not correlated. This allowed us to test for the independent contributions of these parameters to RT.

Before starting the experiment, participants had a short practice session of 40 trials. The mean movement time during this practice session was recorded to calculate the number of rewarded trials in the first experimental block. The data from the practice session were otherwise not further analyzed.

### Data analysis

Reaction times were calculated as the time at which the mouse speed first increased above zero after the go signal. Because of the screen refresh rate (60 Hz), RTs were obtained with a relatively low temporal precision, of one sample every ∼16.7 ms. Trials with RTs under 100 ms were rejected, as potentially anticipatory. In the same way, trials with RTs longer than 1000 ms were rejected. Movement times were calculated as the time taken to click within 20 pixels of the number target, relative to the Go signal. Therefore, movement times included RTs.

To calculate the relationship between RT and response set size, linear regressions were obtained for each participant’s data, and the slopes analyzed RTs were fitted with a linear function, rather than the logarithmic relation normally used for Hick’s Law, for several reasons. First, RTs increase with increasing response set sizes, but a strict logarithmic relationship has not been tested (Lau et al., 2004; Van Eimeren et al., 2006; Kühn et al., 2009; Zhang et al., 2012). Second, the response set sizes considered here (1–4) would fall on the rising arm of the logarithmic function, so could be approximated linearly. Finally, our aim was to establish whether RTs were affected by either initial or final response set sizes, regardless of the precise form of the relationship.

## Results

Participants made few omission errors in *instructed* trials. There was a mean omission rate of 0.94 ± 0.3%. After rejection of omission trials, an average (±SD) of 114 ± 2 trials were included in the *instructed* condition, 186 ± 5 trials in the *no change* condition, 126 ± 17 trials in the selected condition and 150 ± 18 trials in the *reselected* condition. The original selection and reselection conditions presented the highest variability in the number of trials across participants because the exact number of trials that fell in each condition depended on each participant’s behavior. Based on the total number of trials and the combination of initial and final response set sizes, the mean expected number of reselection trials was 139, comparable to the figure obtained.

Trials with RTs shorter than 100 ms were rejected, as potentially anticipatory. Overall, 26 ± 24% trials were rejected, across all participants and conditions. The high number of mean rejected trials was mainly driven by two participants who had a strong tendency (>60% of trials) to anticipate their movements to the Go signal. The results reported here remained valid when we excluded the data from these participants from the analysis.

Differences between the proportions of rejected trials were examined. A two-way 4 × 2 repeated measures ANOVA with the factors of condition and ET revealed significant differences between conditions (*F*_3,48_ = 48.16, *p* < 0.001). The highest proportion of rejected trials due to anticipation was in the *instructed* condition, where participants knew that the instructed target (“5”) would not disappear. There were no significant differences between the proportion of rejected trials in the critical *selected* and *reselected* conditions (*F*_1,16_ = 0.98, *p* = 0.338). Average numbers of trials are shown in Table 1.

**Table 1:** **Final mean (±SD) number of trials per condition after rejecting incorrect and anticipatory trials**.

Condition	*Instructed*		*Original selection*		*Reselection*		*No change*	
ET	Short	Long	Short	Long	Short	Long	Short	Long
Mean number of trials (±SD)	38 ± 16	38 ± 15	58 ± 22	57 ± 20	48 ± 17	47 ± 17	69 ± 22	69 ± 25

### Conditions with no changes in set size

**Figure 4:**
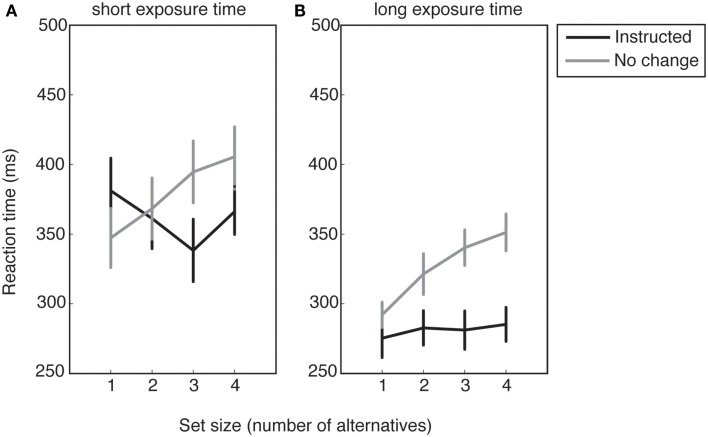
**Reaction times as a function of response set size for (A), short exposure time trials and (B), long exposure time trials**. Error bars show standard error of the mean.

In trials in which no numbers disappeared from the initial response set, initial and final response sets were equivalent, so the only factors of interest were condition (*instructed*/*no change*) and ETs (short/long). The RT averaged across all participants for each response set size is shown in Figure 4.

To explore the effect of ET and voluntary selection, we obtained the mean RTs collapsed across all response set sizes for each condition (Figure 4). A 2 × 2 ANOVA with the factors of condition (*no change*/*instructed*) and ET revealed a main effect of ET (*F*_1,16_ = 22.26, *p* < 0.001), suggesting that participants prepared their motor response during the ET.

There was also a significant main effect of condition (*F*_1,16_ = 27.02, *p* < 0.001), and a significant interaction effect (*F*_1,16_ = 11.01, *p* = 0.004). Follow-up *t*-tests revealed a significant difference between the *instructed* and *no change* conditions for short ETs (*t*_16_ = −2.14, *p* = 0.048), and a strongly significant difference for long ETs (*t*_16_ = −8.73, *p* < 0.001). The longer RTs for the *no change* condition compared to the *instructed* condition reveal an RT cost for voluntary action selection in the former.

To test the effects of set size on RTs, we fitted linear regressions to each participant’s data in each cell of the design, and performed a two-way repeated measures ANOVA with factors of condition (*no change*/*instructed*) and ET (short/long) on the estimated slope parameters (see Figure 4).

Results revealed a main effect of condition (*F*_1,16_ = 14.28, *p* = 0.002) but no significant main effect of ET (*F*_1,16_ = 0.09, *p* = 0.773) or interaction effect (*F*_1,16_ = 0.14, *p* = 0.709). The significant main effect of condition was expected, and consistent with Hick’s law. Whereas voluntary response selection amongst larger sets should have an RT cost in the *no change* condition, the response set size should have no effect on instructed RTs, since there is no selection process other than visually searching for the target.

### Conditions with different initial and final response sets

**Figure 5:**
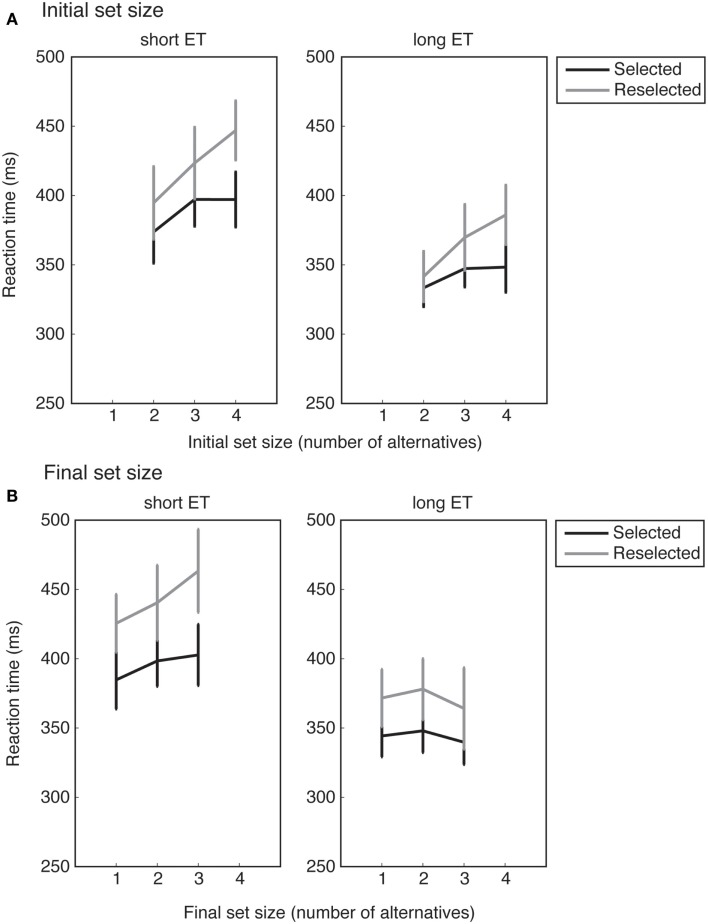
**RTs averaged across all participants**. **(A,B)** show the mean RTs as a function of the initial and final response set sizes, respectively. For long exposure time (ET) trials, RTs increase monotonically with the *initial* response set size, but not with the *final* response set sizes. Error bars show standard error of the mean.

In the *selected* and *reselected* conditions, one or more numbers were removed from the response set. Consequently, the initial and final response set sizes differed. RTs as a function of either the initial set size or final response set sizes are shown in Figure 5.

Our design carefully ensured that the initial and final response set sizes were not correlated. For example, trials with an initial response set of four could have any of the possible final response set sizes of 1, 2, 3, or 4. Similarly, trials with an initial response set of 3 could have any of the possible final response set sizes of 1, 2, or 3. Therefore, the relationship between mean RT and the size of the initial response set could potentially differ from the relationship between the mean RT and the size of the final response set. We could use this design feature to investigate whether the internal representation was updated to match the final set size. Updating the internal representation predicts a stronger relation between RT and final set size than between RT and initial set size, while failure to update predicts the opposite pattern.

To examine the effects of reselection on RTs, we first analyzed the mean RTs, irrespective of set size, by incorporating the factor of response set into a 2 × 2 × 2 ANOVA with the factors of ET (short/long), condition (*selected*/*reselected*), and set (initial/final). There was a main effect of ET (*F*_1,16_ = 39.24, *p* < 0.001), suggesting that long ETs allowed for stronger motor preparation than shorter ETs, and validating the ET manipulation. We also found a significant main effect of condition (*F*_1,16_ = 14.06, *p* = 0.002). We interpret this as an RT cost of the inhibition of the original action plans and the process of number reselection.

We also found a main effect of response set (*F*_1,16_ = 6.33, *p* = 0.023), with larger response sets generally associated with larger RTs. There was a significant response set × ET interaction (*F*_1,16_ = 9.54, *p* = 0.007). There were no other significant effects.

**Figure 6:**
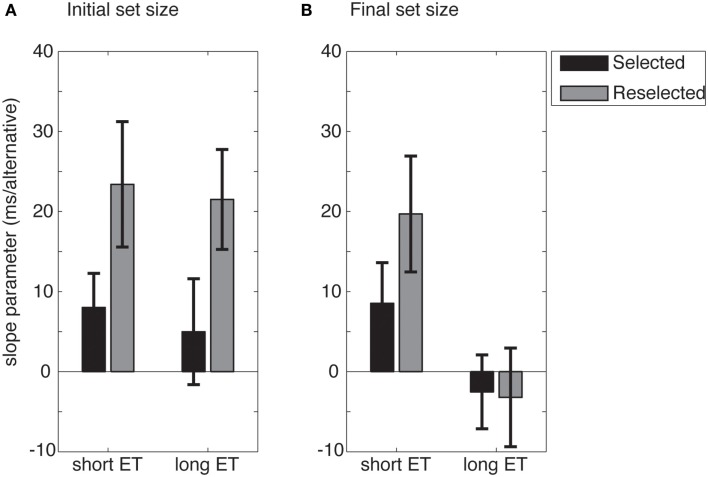
**Mean slope of the linear fit to the RTs as a function of response set size, for either initial (A) or final response set sizes (B)**. For long exposure time (ET) trials, RTs increase as a function of the initial, but not the final, response set size. Error bars show standard error.

More importantly, we analyzed the slopes of the individual linear fits for the RTs in a repeated measures 2 × 2 × 2 ANOVA. Differences between the slopes allowed us to infer whether an updating of internal representation did or did not occur when disappearance of an item from the initial response set triggered reselection. The mean slope estimates for the *selected* and *reselected* conditions are shown in Figure 6.

Results from the three way ANOVA revealed a significant main effect of ET (*F*_1,16_ = 6.87, *p* = 0.019), presumably also revealing the results of increased motor preparation.

Importantly, we also found a main effect of response set (*F*_1,16_ = 5.12, *p* = 0.038), and a significant response set × ET interaction (*F*_1,16_ = 6.551, *p* = 0.021). This indicates that the initial response set size had a stronger impact on RTs than the final response set size. No other effects were significant.

To investigate the response set × ET interaction, the slope estimates were collapsed across conditions. Follow-up *t*-tests revealed no differences between initial and final response set sizes in the short ET conditions (*t*_16_ = 0.28, *p* = 0.779), but clearly significant differences between the initial and final response set sizes in the long ET conditions (*t*_16_ = 4.04, *p* < 0.001). When participants had enough time to represent the initial response space and prepare actions (in long ET conditions), the number of initially available response alternatives seem to have a measurable effect on RTs, even if the selected alternative later became unavailable. This suggests that the internal representation of the response space, once it is built, is not fully updated if the number of response alternatives is reduced. That is, the internal representation of the response space displays persistence.

### RTs as a function of the binary logarithm of the response set size

We obtained the above results by estimating linear fits of the RTs as a function of the different response set sizes (see Materials and Methods). As a control, we also explored whether the same results would be valid if the RTs were described as a function of the binary logarithm of response set size, as established by Hick’s law (Hick, 1952). Because a maximum of four response set sizes are not enough to produce reliable estimates of the parameters of a logarithmic function, we considered the linearized response set size. In other words, we conducted the same analyses, but considering RTs as a function of the binary logarithm of the response set size, rather than as a function of the response set size itself. This analysis yielded similar results as the ones reported above.

A 2 × 2 × 2 ANOVA on the slopes of the RTs as a function of the binary logarithm of the response set size revealed a main effect of response set (*F*_1,16_ = 9.17, *p* = 0.008), a marginally significant effect of ET (*F*_1,16_ = 4.41, *p* = 0.052), and a marginally significant effect of condition (*F*_1,16_ = 4.41, *p* = 0.051). There was a trend for a significant response set × condition interaction (*F*_1,16_ = 3.99, *p* = 0.06). No other effects were significant.

Finally, in the analysis reported above, we calculated RTs as the first point in time at which the speed of the cursor was non-zero. To ensure that the obtained results were not an artifact of the way in which the RTs were defined, we performed the same analysis on the slopes of the linear fits in two alternative ways. First, we calculated RTs as the time at which the cursor had covered 25% of the total distance in each trial. Second, we performed the same analysis on movement times, calculated as the time to click on the final target. In both cases, the three way repeated measures ANOVA yielded a significant effect of response set (*F*_1,16_ = 12.8, *p* = 0.003 and *F*_1,16_ = 13.32, *p* = 0.002, respectively).

In sum, the main effect of response set size remained after addressing the relationship between RTs and response set sizes in a way that followed more strictly the formulation of Hick’s law. The effect was not highly sensitive to the way in which the RTs were calculated.

## Discussion

In this study we aimed at answering the question of whether selected response alternatives that are no longer available in the environment nevertheless remain represented in the brain. The internal response sets driving RTs corresponded more closely to the initial than to the final external response sets. This suggests that the internal response sets are in fact resilient to external change, and “lag behind” sudden changes in the external environment.

### Conditions with equivalent initial and final response set sizes: Instructed and no change

We first compared the *no change* and *instructed* conditions, where the initial and final set sizes were indistinguishable. Whereas the *no change* condition required intentional response selection, the *instructed* condition required only visual search to identify the instructed target. The *no change* condition was informative of the relationship between the RTs and the response set size. RTs in the *no change* condition showed a positive linear relation with response set size. Conversely, RTs in *instructed* trials did not depend on the response set size (i.e., the estimated slopes of the linear trends did not differ significantly from zero). This may seem surprising, as monotonic increases in instructed RTs as a function of response set size have been well documented (Hick, 1952). In this experiment, however, the ET temporally separated the processes of visual search and action initiation. This may explain the null effect of response set size on instructed RTs. Importantly, this validates the ET manipulation, aimed at allowing for selection and motor preparation, and suggests that the results cannot be easily explained by visual search processes.

We analyzed the effects of ET (short *vs*. long) and condition (*instructed* *vs*. *no change*). Shorter ETs were associated with longer mean RTs and with steeper dependencies of RTs on response set size. This suggests that longer ETs allowed for movement preparation, reducing the mean RT and decreasing the impact of increasing the number of response alternatives.

### Conditions with unequal initial and final response set sizes: “Original selection” and “reselection”

In the selection and reselection conditions, some target numbers disappeared from the initial response set. Because the initial and final response set sizes were not correlated, we incorporated them as independent factors in statistical analyses.

In both *selected* and *reselected* conditions, trials with longer ETs showed shorter RTs. This effect mirrors what was found in the *no change* and *instructed* conditions, and once again suggests that response selection and motor preparation took place during the ET. Further, as expected, longer RTs were found in reselection trials due to the cost of response inhibition and reselection.

Crucially, an analysis of the slopes of the linear fits revealed stronger dependencies of the RTs with initial response set sizes as compared to final response set sizes. This suggests that the initial response set size had a stronger influence on the RTs than the final response set size. This effect was strongest particularly for long ET conditions. Longer ETs may allow for stronger and more stable encoding of the initial response set size, leading to more persistence of the internal representation of the initial response set.

A comparison of the *selected* and *reselected* conditions revealed that reselection processes led to longer RTs, in all response set sizes. This is consistent with an RT cost of abandoning the initially selected response alternative and selecting a new one.

Interestingly, however, the persistence of the response space was not directly related to the disappearance of the selected alternative itself. The comparison between the *selected* and *reselected* conditions did not reveal differences in the RT slopes. This suggests that the persistence of the initial set is not uniquely driven by the disappearance of the selected alternative. Instead, these results suggest that it is the non-specific encoding of the entire response set that makes it persistent in face of external change.

This effect recalls Schacter and Addis (2007) hypothesis, from the very different field of episodic memory, that the brain flexibly recalls all past events, in order to constructively simulate and prepare for future events. Over a shorter time scale such as our task, recollection of all possible past experiences of action selection, could speculatively take place in the context of working memory.

Intriguingly, a marginally significant effect of condition on the RT slopes was found when the binary logarithm of the response set size was considered instead of the absolute response set size. This analysis was motivated by exploring a strict implementation of Hick’s law, which establishes that instructed go RTs vary linearly with the binary logarithm of the response set. However, there is no solid empirical evidence for such a strict implementation of Hick’s law, so the potential effects of intentional selection remain speculative.

Additional controls showed that the significant effect of response set size was not an artifact of the way in which RTs were measured. Two additional controls considered complete movement times, or measured RTs as the time at which the distance traveled by the cursor was 25% of the final distance. In both cases, a statistically significant effect of response set size was found.

### Persistent representations of voluntarily selected response alternatives

We have previously argued that the distinction between externally instructed and internally driven action systems (Krieghoff et al., 2011) is also applicable to action inhibition (Filevich et al., 2012). In other words, we suggest that neural systems for externally instructed inhibition (as in the case of stop signal tasks) do not fully overlap with those for internally driven action inhibition. Moreover, the two systems may differ quantitatively as well as qualitatively: intentional decisions for both action and inhibition may have a weaker neural signal, or lower levels of evidence, than their instructed counterparts (Fleming et al., 2009; Filevich and Haggard, 2012).

The present experiment revealed a process of inhibition and subsequent reselection for intentional actions. How do these processes fit with the intentional/instructed distinction mentioned above? First, the initial selection of a number to which to move was intentional, in the sense of internally generated rather than externally triggered. The inhibition of the action was achieved by removal of the selected item. This seems to have some features in common with intentional inhibition, such as the absence of an overt stop signal, but some features in common with external inhibition, since there is an environmental change that triggers inhibition. Our analyses focused on the persistence *vs*. flexible updating of an internal representation of the response space for intentional action selection.

Our result seems relevant to the previously introduced concepts of parallelism and strength of evidence in action selection, in two ways. First, persistence of action representations is naturally linked to parallel action preparation. If an intentionally selected action is not removed when the response set is updated, it will potentially remain a candidate for selection, and may competitively interfere with subsequent action selection processes. Second, since the to-be-inhibited item apparently remained in the internal representation of the response space, we might conclude that the processes of intentional inhibition are relatively weak.

We could not directly compare flexibility of the internal representation of response spaces for internally driven and externally instructed selection, because we did not remove any numbers from the initial response set in *instructed* trials. Indeed, removing options in an *instructed* condition would be meaningless. If the removed item were different from the instructed item, then no inhibition would be expected, and if the removed item was the instructed item, the task effectively becomes a NoGo task rather than an instructed action task. Rather, we used our *instructed* condition as a baseline for modeling the relationship between RTs and set sizes. Therefore, we cannot directly compare the persistence of action alternatives between our intentional selection and an externally triggered alternative.

Neurophysiological data suggest that in cases of active maintenance of multiple response alternatives (in this case, selected and non-selected), all representations are scaled down in proportion to the total number of active representations. For example, in a saccade-to-target experiment, Purcell et al. (2012) found that firing rates in monkeys’ visual and motor areas decreased monotonically with increasingly larger response sets (two, four, or eight total items).

The results from Purcell et al. (2012) provide a plausible neural explanation for Hick’s law. Larger response set sizes will set a lower baseline firing rate from which perceptual evidence needs to be accumulated until it reaches a decision threshold (Gold and Shadlen, 2001; Smith and Ratcliff, 2004). In turn, this may translate into longer accumulation times, manifested as longer RTs.

Accumulator models, traditionally restricted to perceptual decision-making, have recently been extended to voluntary choices in human behavior (Zhang et al., 2012). These, “voluntary” accumulator models, analogous to perceptual models, may relate to the present findings. Different neuronal assemblies may gather “voluntary” information for each target. Speculatively, spiking activity in neuronal assemblies that correspond to the alternatives that are no longer available may not be fully inhibited immediately after target disappearance. In line with the results reported by Purcell et al. (2012), initial firing rates of each neural assembly may be lower for larger initial response set sizes in this experiment, leading to longer intentional RTs.

Together, these results suggest an interesting corollary of the relative weakness of internally driven decisions mentioned earlier (Fleming et al., 2009; Filevich and Haggard, 2012). Weak internal decisions may set lower baseline firing rates for the representations of each of the potential response alternatives. In turn, these weak representations may not efficiently inhibit the representations of the unavailable response alternatives.

## Conclusion

Our findings suggest that persistence of unselected options in the internal response space could be one reason why people often feel they “could have done otherwise.” This feeling may be sufficient to generate a feeling of freedom, even when the current external environment in fact limits what an agent can do.

Several important mental health disorders can be linked to counterfactual representation, including frustration, (expected outcomes of chosen actions are not obtained), regret (desire of having selected the alternative response alternative), and rumination (persistence of these feelings across time). These processes and states all require a continued representation of action alternatives that are not actually available. Previous investigations of these concepts have been limited by the difficulty of relating them to control of actual behaviors. Our concept of persistent internal representation of items in the response space may offer a window into understanding these processes.

## Conflict of Interest Statement

The authors declare that the research was conducted in the absence of any commercial or financial relationships that could be construed as a potential conflict of interest.
